# Medical Ozone Increases Methotrexate Effects in Rheumatoid Arthritis Through a Shared New Mechanism Which Involves Adenosine

**DOI:** 10.3390/ijms26115256

**Published:** 2025-05-29

**Authors:** Olga Sonia León Fernández, Gabriel Takon Oru, Renate Viebahn-Haensler, Gilberto López Cabreja, Irainis Serrano Espinosa, María Elena Corrales Vázquez

**Affiliations:** 1 Pharmacy and Food Institute, University of Havana, Havana 13603, Cuba; orugabriel@yahoo.com; 2 Medical Society for the Use of Ozone in Prevention and Therapy, D-76473 Iffezheim, Germany; renateviebahn@t-online.de; 3 National Institute of Rheumatology, Ministry of Public Health, Havana 19210, Cuba; gilberto.lopez@infomed.sld.cu (G.L.C.); serranoespinosairainis@gmail.com (I.S.E.); 4 Clinical Laboratory of the Surgical and Clinical Teaching Hospital “10 de Octubre”, Faculty of Medical Sciences, Ministry of Public Health, Havana 19210, Cuba; mariaelenacorralesvazques@gmail.com

**Keywords:** ozone, reactive oxygen species, rheumatoid arthritis, methotrexate, A_1_ adenosine receptors

## Abstract

Medical ozone is a redox regulator with beneficial effects in oxidative etiology diseases such as rheumatoid arthritis (RA). The aim of this study is to conduct a holistic review of different pharmacological trials involving ozone in model diseases, as well as the clinical responses of RA patients. The ROS (reactive oxygen species) involved in RA and their relationship with the main pathological pathways of this autoimmune disease are considered here. The integrative analysis of experimental results from animals with clinical findings reveals that both methotrexate (MTX) and medical ozone share common mechanisms via adenosinergic regulation. This finding enables us to propose a new pharmacological mechanism in the treatment of RA. We conclude that MTX + medical ozone combined therapy reduces ROS overproduction and the generation of proinflammatory cytokines and decreases anti-cyclic citrullinate peptide levels by a mutual mechanism involving adenosine A_1_ receptors.

## 1. Introduction

The loss of cellular redox balance leads to oxidative stress (OS) as a result of excessive ROS production that exceeds antioxidant defenses [1] and causes cellular damage with a loss of vital functions.

It is important to note that OS does not always promote damage. Some ROS participate in signaling mechanisms and regulate processes essential for life. A good example is nitric oxide, a free radical and vasodilator, released by endothelial cells; its deficit is associated with cardiovascular and other diseases. OS is involved in a variety of autoimmune diseases of oxidative etiology [2,3], particularly rheumatoid arthritis (RA). RA is a chronic systemic autoimmune disease causing progressive disability and premature death [4]. It is a symmetric peripheral disease involving bone erosion, proximal involvement, and destructive bone lesions. In addition to this, RA patients display synovitis, morning stiffness, and/or immobility of their proximal interphalangeal joints [5]. Finally, such patients suffer joint failure due to cartilage damage and severely weakened tendons and ligaments [6].

In the development of RA, a proinflammatory and hypoxic scenario is generated in the synovial tissue, which leads to the overproduction of reactive oxygen species (ROS) with DNA damage and mitochondrial dysfunction. The synovium is the principal target, as synovitis is associated with the degree of RA activity. This compartment is severely infiltrated by immune system cells, leading to neovascularization. The joints of patients affected by RA show inflammation, edematous synovial tissue with hyperemia, and other alterations [7].

Medical ozone consists of an ozone/oxygen mixture at a concentration between 0.05 and 5 volume percent. It is generated by electrosynthesis from a stream of pure oxygen and is used in medicine [8].

Studies conducted on cells [9], animal models of disease [10,11], and controlled clinical studies [12,13] have demonstrated the efficacy of medical ozone. Its administration at low doses and with a number of controlled treatments represents an effective and safe complementary/basic therapy. The pharmacological mechanism of action of medical ozone is essentially the regulation of cellular redox status, which has been demonstrated [14] and recognized by various authors [15,16,17]. Ozone stimulates antioxidant defense systems, reducing cellular damage, expressed through a reduction in injury markers and the preservation of target organ morphology. The antioxidant effects of ozone are associated, among other factors, with its ability to activate the nuclear transcription factor Nrf2 (erythroid-derived 2)-like 2 (Nrf2). The stimulation of antioxidant response gene (ARE) recognition leads to an increase in reduced glutathione (GSH), glutathione peroxidase (GSH-Px), superoxide dismutase (SOD), and other antioxidant systems [18].

RA is a multisystem autoimmune disorder conditioned by various factors. This characteristic may explain why the drugs for its treatment have different mechanisms of action and have been grouped under the generic name of Disease-Modifying Antirheumatoid Drugs (DMARDs). This heterogeneous pharmacological group is classified into conventional synthetic DMARDs (csDMARDs), biological DMARDs (bDMARDs), and targeted synthetic DMARDs (tsDMARDs). csDMARDs are recommended as a first-line therapy for patients with an early diagnosis of RA, while bDMARDs and tsDMARDs are used when there is therapeutic failure or intolerance to csDMARDs [19].

OS is closely associated with the pathophysiology of RA and, with the exception of natural drugs, including ozone, and a few conventional and biologic agents, most DMARDs do not have the control of ROS and cellular redox balance among their therapeutic targets [20]. ROS are involved in important functions and mechanisms associated with both the innate and acquired immune responses [3].

**Figure 1:**
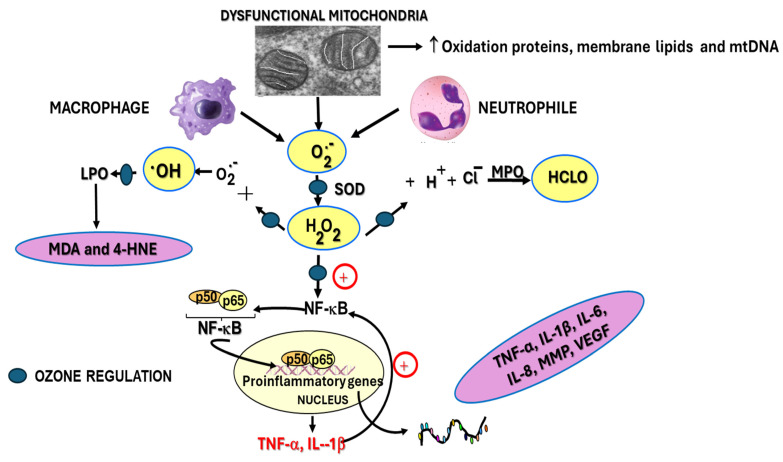
Contribution of ROS to trigger rheumatoid arthritis (RA) disease and its progession. Dysfuncional mitochondria, macrophages and neutrophil release superoxide radical which is the precursor of the most ROS and is metabolized by superoxide dismutase activity to hydrogen peroxide. This ROS unchain the generation of different ROS such as hydroxy radicals, activates NF-κB and produce hypochlorous acid. All of them are close associated to inflammation, cartilage damage and bone erosion in RA. Superoxide radical O_2_^−^; H_2_O_2_; hydrogen peroxide; OH^−^; hydroxyl radical; HCLO, hypochlorous acid.

Figure 1 shows the main reactive oxygen species (ROS) involved in different events associated with the development and progression of rheumatoid arthritis (RA), including superoxide radicals, hydrogen peroxide, hydroxyl radicals, and hypochlorous acid [21]. Medical ozone is able to neutralize the harmful effects of these ROS by increasing antioxidant defenses, primarily GSH (reduced glutathione), SOD (superoxide dismutase activity), and CAT (catalase activity), which are involved in metabolism and the restoration of hydrogen peroxide levels. This prevents the injury cascade that leads to the generation of MDA and 4-HNE (malondialdehyde and 4-hydroxynonenal, respectively), proinflammatory cytokines, and the chlorination of essential biomolecules.

Conventional DMARDs and biologics are effective, but can also result in adverse reactions that can lead to treatment discontinuation. For conventional DMARDs, these include gastrointestinal, hepatic, hematologic, and pulmonary disorders—consisting of diarrhea, nausea, liver damage with a lower incidence of cirrhosis, thrombocytopenia, leukopenia, pulmonary fibrosis, and pneumonitis [22]. Biologics, which are still considered promising, are prone to cause infections due to their suppressive effects on the immune system, which can become severe when the dose is increased. Several examples demonstrate this. A safety and efficacy study on etanercept (25 mg twice/week for 10 years) in 1272 patients with RA reported 5 opportunistic infections, 29 cases of sepsis, 14 lymphomas, and 61 deaths, but the occurrence of serious adverse events was higher in longstanding RA patients than in early RA patients [23]. TNF-α inhibitors increase the risk of developing tuberculosis (TB). The use of these inhibitors was associated with an 18-fold increase in TB incidence in RA patients from a high-incidence area [24].

Among the conventional DMARDs, methotrexate (MTX) stands out and is considered to be a first-line drug for the treatment of RA. The participation of adenosine has an important significance for the therapeutic actions of MTX in the treatment of patients with RA. It has been recognized that adenosine acts as an immunosuppressant that contributes to strengthening immunological tolerance [25].

Ozone is an effective and safe therapeutic option for the treatment of RA. Ozone has been used in medical practice since 1915 [26] in thousands of patients. Its distinctive features, with respect to DMARDs, are the regulation of cellular redox balance and greater safety, as no adverse reactions have been found [27,28], although work continues on the observation of undesirable reactions, in a specific clinical order.

The aim of this review is to present a holistic analysis of a selection of redox results from our previous studies on medical ozone in disease and clinical models (Table 1). In this review, the interrelation between animal studies and clinical responses in patients with RA is discussed. An integrative analysis leads to the proposal of a new mechanism of action. We conclude that the improvement observed in the clinical response of patients with RA treated with the combined therapy methotrexate + medical ozone, compared to monotherapy, is the result of a common mechanism of action, shared by methotrexate and medical ozone, with the participation of adenosinergic transmission.

## 2. Methods

All studies presented in this review were obtained using an OZOMED brand ozonizer, manufactured by the National Center for Scientific Research (CENIC), Cuba. Ozone—obtained from medical-grade oxygen—was used immediately as generated, hereby representing only about 5% of the gas (O_2_/O_3_) mixture. Rectal insufflation was selected as the administration route. The ozone concentration was measured by using a built-in UV spectrophotometer at 254 nm (accuracy: 0.002 at 1 absorbance unit, repeatability: 0.001 absorbance unit, and calibrated with internal standard). The ozone dose is the product of the ozone concentration [expressed as mg/mL by the gas (O_3_/O_2_) volume]. By knowing the body weight of the rat/mouse, the ozone dose was calculated as mg/kg as in our previous papers [10]. Rats (Wistar or Lewis)/mice (BALB/c) received 10–15 ozone treatments, one per day at an ozone concentration of 50 µg/mL. BAlB/c mice. Wistar rats are widely used species in different pharmacological models. The chronic RA model, induced by SCW (streptococcal cell wall)-induced chronic inflammatory arthritis, requires the use of Lewis rats, since the disease is only expressed in this lineage. Preclinical studies were aimed at investigating the effects of ozone on RA in two models that would demonstrate whether ozone “per se” had effects on any of the pathological events of RA. Furthermore, given the complexity of this autoimmune dysfunction, it was advisable to evaluate at least two different models [35]. The selected models had to comply with established concepts of pharmacology. Thus, the following were chosen: (1) the acute, i.e., prophylactic model, in which the carrageenan-induced synovitis model was used [36], and (2) the chronic model, i.e., the therapeutic effect. The streptococcal-cell-wall-induced chronic arthritis model was selected [37].

In clinical studies (randomized, controlled, and informed-consent-based efficacy trials), rectal insufflation and ozone concentrations from 25 mg/L to 40 mg/L were used in stepped application and in increasing order, as follows: 1st week: 25 mg/L, 100 mL; 2nd week: 30 mg/L, 150 mL; 3rd week: 35 mg/L, 200 mL; and 4th week: 40 mg/L, 200 mL. Patients received 20 treatments (five per week, Mondays through Fridays).

### Statistical Analysis

For preclinical studies, a preliminary statistical test was applied to detect outliers (OUTLIERS). A one-way ANOVA test was then used, followed by a test of homogeneity of variance (Bartlett–Box). A multiple comparison test (Duncan test) was also used. Experimental data are expressed as the mean ± standard deviation of 5 animals. The level of statistical significance was at least *p* < 0.05. Data processing was performed using a database and the SPSS 22 statistical package. For clinical studies, canonical discrimination analysis was used to identify variables capable of distinguishing changes in RA patients (MTX or MTX + ozone medium). Comparisons of each variable (before the start and at the end of the study) for each treatment were evaluated using the Wilcoxon rank test and the Student’s *t* test for correlated samples. To compare each variable at the end of the prospective study with respect to treatment, the Mann–Whitney test and the Student’s *t* test for independent samples were used. The risk of alterations in GGT and ALP activity at the end of the study and for each treatment was estimated for GGT using the Cochran and Mentel/Haenszel tests. For ALP, the relative risk (RR) or odds ratio was calculated. The level of statistical significance was at least *p* < 0.05. The experimental data are expressed as mean ± standard deviation. Data processing was performed using a database and the SPSS statistical package.

## 3. Results

Medical ozone, a regulator of redox targets with an impact on RA.

### 3.1. Studies in Animal Models

Medical ozone is an ozone/oxygen mixture (O_3_/O_2_) obtained by electrosynthesis by passing a flow/stream/specific volume of pure O_2_ through a silent electrical discharge generated by specific equipment designed for such purposes, obtaining an ozone concentration between 0.05 and 5 volume percent [8].

The mechanisms associated with the therapeutic effects of ozone are based on its ability to regulate cellular redox state. The protective actions of ozone are similar to those described for Ischemic Preconditioning, which refers to the phenomenon in which repeated short episodes of ischemia/reperfusion (2–3 min) protect the myocardium against damage from sustained ischemia/reperfusion (lasting hours) [38]. Analogous to the concept of Ischemic Preconditioning, it is proposed that ozone—a potent oxidizing agent—when administered in low doses and under controlled treatment conditions, induces mild oxidative stress that triggers antioxidant defense mechanisms and regulates oxidative stress, which is implicated in a variety of diseases with oxidative etiology. This response has been confirmed in subsequent studies and has been termed “Ozone Oxidative Preconditioning (OzoneOxPre)” [14].

Indeed, medical ozone adheres to the principle of hormesis—low concentrations (or doses) exhibit a high efficacy, which decreases with increasing concentrations and may eventually become questionable or even toxic. The dose–response relationship for the systemic application of ozone—administered as standardized major ozone autohemotherapy or rectal ozone gas insufflation—results in the following effective concentration range: 10–40 µg ozone/mL of the ozone/oxygen mixture is considered as physiologically effective and is recommended for systemic use. In contrast, higher concentrations ranging from 60 to 100 µg/mL exhibit an antimicrobial effect and are widely used for the treatment of infected wounds, diabetic foot, and pressure ulcers [39]. The antioxidant protective effects of ozone are mediated by the activation of Nrf2 [18], the regulation of NF-κB [31], and potentially other mechanisms.

Studies carried out in different disease models using experimental animals have shown that medical ozone, per se, is capable of regulating cellular redox state by acting on different processes involved in RA—this is referred to in Figure 1.

**Figure 2:**
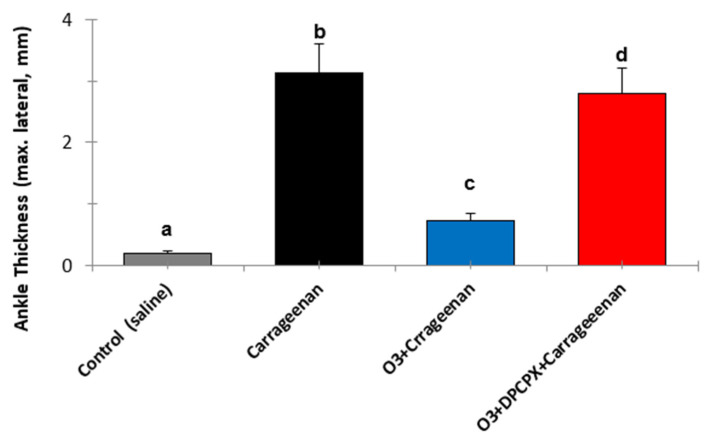
The effects of medical ozone on carrageenan-induced knee inflammation in the acute model with BALB/c mice, 4 h after carrageenan administration. Data represent the mean ± SD of each group (*n* = 5). The different letters indicate significant differences (*p* < 0.5) between groups [29].

**Figure 3:**
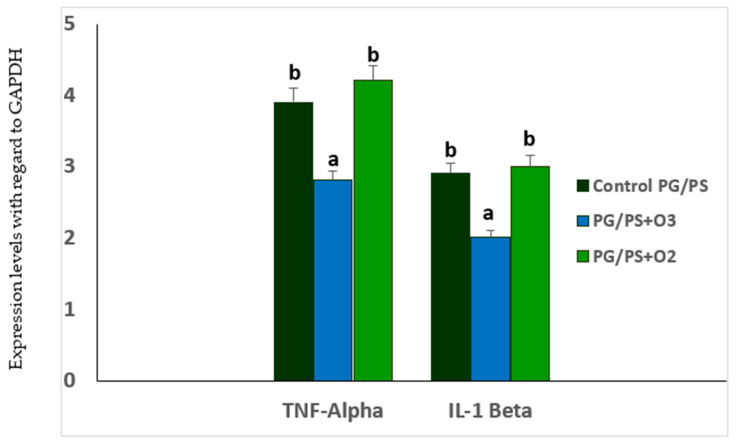
TNF-α and IL-1β mRNA levels in spleen homogenates of Lewis rats in the PG/PS-induced arthritis model and the influence of ozone/oxygen treatments. GAPDH = glyceraldehyde-3-phosphate dehydrogenase. Data represent the mean ± SD of each group (*n* = 5). The different letters indicate significant differences between groups (*p* < 0.05) [30].

In acute (carrageenan-induced synovitis) and chronic models through the administration of a fraction of the streptococcal wall, polyglycan/polysaccharide peptide (PG/PS), the beneficial effects of medical ozone were demonstrated by reductions in inflammation and pain in the acute model [29] (Figure 2). Likewise, in the chronic model (Figure 3), the levels of mRNA transcripts for TNF-α and Il-1β were reduced in the group treated with ozone when compared to those in the control group, which had been treated with the inducer PG/PS and the PG/PG + oxygen group (ozone vehicle) [30].

It is evident how medical ozone, administered by rectal insufflation, prevented carrageenan-mediated inflammation (0.73 ± 0.04 vs. 3 ± 0.5 mm, respectively) without differing significantly from the control (saline) (0.2 ± 0.08). The participation of adenosine A_1_ receptors in the anti-inflammatory actions of ozone was demonstrated when DPCPX (8-cyclopentyl-1,3-dipropylxanthine), a specific antagonist of these receptors, blocked the protective effects of medical ozone by reestablishing the inflammatory process (3 ± 0.5 vs. 2.8 ± 0).

The participation of adenosine has an important significance in the therapeutic actions of methotrexate (MTX) in the treatment of patients with RA. Adenosine is considered to be one of the probable mechanisms of action of MTX, due to its ability to inhibit the release of proinflammatory cytokines [40]. The beneficial effects of ozone, mediated by A_1_ adenosine receptors, have been demonstrated in different disease models—one example is ischemia/reperfusion injury of the liver. In this model, medical ozone was shown to promote adenosine accumulation by regulating the activity of adenosine deaminase, the enzyme responsible for its degradation to inosine [32]. In another experiment, ozone reduced the time up to the first seizure in pentylenetetrazol-induced seizures [41]. Similar results were achieved when carrageenan-induced synovitis was studied in mice [36].

The expression of A1 adenosine receptors (A1AR) is redox-dependent. The protective effects of Ozone Oxidative Preconditioning (OzoneOxPre), mediated by A1AR, may result from transient oxidative stress. Supporting this hypothesis, Nie et al. [42] demonstrated that oxidative stress regulates the expression of the A1AR subtype. In their study, oxidative stress induced by certain antineoplastic agents and hydrogen peroxide (H_2_O_2_) upregulated A1AR expression in hamster ductus deferens smooth muscle cells. Specifically, treatment with cisplatin—a chemotherapeutic agent known to enhance reactive oxygen species (ROS) production—resulted in an approximately two-fold increase in A1AR levels, as measured by binding assays using the antagonist radioligand DPCPX and the agonist radioligand ^125^I-N^6^-2-(4-amino-3-phenyl)ethyladenosine (APNEA) and confirmed by Western blot analysis.

Other ROS inducers, including daunorubicin, doxorubicin, mitoxantrone, and H_2_O_2_, also effectively increased A1AR expression. Furthermore, another study demonstrated that the ROS-mediated upregulation of A1AR was attenuated by catalase—a hydrogen peroxide scavenger—thereby reinforcing the role of oxidative stress in modulating A1AR expression [34].

Figure 3 shows the mRNA levels for the proinflammatory cytokines TNF-α and IL-1β, which, together with IL-6, are characteristic of the cytokine storm in RA. Table 2 shows both means and 95% Intervals of Confidence for the mRNA levels of both cytokines.

Medical ozone significantly reduced the concentrations of both cytokines that are generated as a result of activation of the nuclear transcription factor NF-kB.

**Figure 4:**
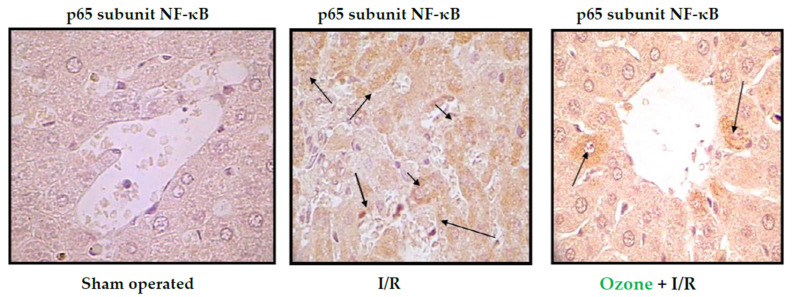
Immunohistochemistry of liver slides. Data shown are representative of at least seven rats. “Sham operated animals” means that the animals were submitted to anesthesia and laparotomy; I/R 90 min of ischemia followed by 90 min of reperfusion; O_3_ + I/R, animals preconditioned with ozone and subjected to I/R. Arrows indicate the reaction intensity between p65 subunit with its antibody [31].

Figure 4 shows the results of immunohistochemistry of the p65 subunit of NF-κB in ischemia/reperfusion (I/R) of the liver.

The p65 subunit of NF-κB is the one that recognizes response elements in DNA and promotes the transcription of proinflammatory cytokines (Figure 1). In Figure 4, intense p65 immunoreactivity was observed in the I/R group, whereas ozone-treated animals showed only scattered zones of reaction. The results suggest that ozone regulates the expression of this factor—among other mechanisms, by its actions on the control of hydrogen peroxide generation (Figure 1), one of the activators of this factor.

Table 3 shows the levels of some redox markers in both models (carrageenan, acute, and PG/PS (chronic) and their Confidence Intervals).

It can be seen how, in the acute synovitis model, ozone restored the concentrations of hydroperoxides to control levels, as well as MDA, the terminal product of lipid peroxidation, which is generated by the formation of hydroxyl radicals through the peroxidation of membrane lipids (Figure 1). MDA has been recognized as a biogenic aldehyde forming adducts with proteins and contributing to the loss of immune tolerance, among other effects [43,44]

In the chronic model (PG/PS), an increase in SOD was observed, capturing superoxide radicals and generating hydrogen peroxide. This ROS was reduced to water and oxygen by an increase in catalase activity that was observed in the chronic model induced by PG/PS. The increase in CAT (391.07 ± 2.6) was probably the result of a compensatory mechanism leading to an increase in SOD (34.9 ± 0.8), which generates hydrogen peroxide.

### 3.2. Studies in Patients with Rheumatoid Arthritis

The treatment options available for RA, where methotrexate (MTX) remains the gold standard for this disease, as well as the so-called biologicals that can reduce pain and stiffness, exhibit a limited effectiveness accompanied by multiple adverse effects. Biological agents (e.g., anti-TNF-α antibodies) are effective, but have adverse effects such as infections in the infusion process and at the injection site, as well as differences in efficacy. With the advent of these new therapies, the treatment of patients with RA has improved. However, due to the heterogeneity and complexity of the pathological mechanisms in RA, some patients still have a poor clinical response, which means that the development of new therapies is still a priority.

The results in patients with RA treated using the combined therapy of MTX + medical ozone were in line with experimental studies. A decrease in swollen and painful joints was observed, as well as an improvement in the performance of daily activities; this included a reduction in the levels of antibodies against cyclic citrullinated peptides and an increase in antioxidant defenses, accompanied by a decrease in redox markers which indicate damage.

Table 4 shows the results obtained after 21 days of treatment in patients treated with MTX and in those who received MTX + medical ozone [33].

At the time of the study, patients in both groups had a disease progression time of 7–11 years and were all receiving MTX (33). The only difference between the two groups was the inclusion of medical ozone in the treatment. No significant difference (*p* > 0.05) between either group was observed for the variables of DAS_28_ and HAQ-DI at the beginning of the study. DAS_28_ decreased significantly (*p* < 0.05) in the MTX + Ozone group, whereas there were no changes in the MTX group. At the end of the clinical study, differences (*p* < 0.05) were observed between both groups. HAQ-DI showed results similar to DAS_28_, and MTX + medical ozone improved the patients’ disabilities, whereas the MTX group ended without changes. Again, significant differences (*p* < 0.05) were found between both groups at the end of the study, showing beneficial results for the group of patients receiving the combined therapy. Both acute-phase reactants (CRP and ESR) decreased (*p* < 0.05) in the MTX + medical ozone group, whereas the MTX group showed no significant changes (*p* > 0.05) at the end of the study.

The MTX + medical ozone group reduced (*p* < 0.05) autoantibodies against cyclic citrullinated peptides, which were not modified (*p* > 0.05) in the group not treated with ozone. Similarly, the autoantibody levels in the MTX + ozone group were lower than those in the MTX group at the end of the study.

**Figure 5:**
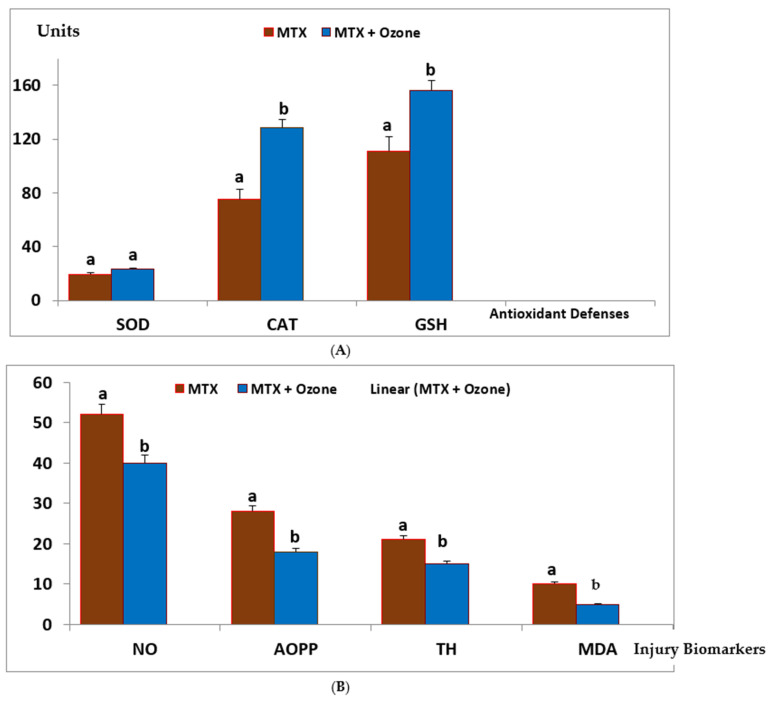
Redox status of RA patients treated with MTX and MTX + ozone combination therapy at the end of the study (24 h after the final medical ozone treatment). (**A**) Antioxidant defense markers and (**B**) injury markers. The units of each marker are SOD (superoxide dismutase activity, U/mL/min), CAT (catalase activity, U/L/min), GSH (reduced glutathione, µM), NO (nitric oxide, µM), AOPPs (Advanced Oxidation Protein Products, µM), TH (Total Hydroperoxides, µM), and MDA (Malondialdehyde, µM). The data represent the mean ± SD of each group. Different letters indicate significant differences (*p* < 0.05) [33].

Plasma biomarkers of protection (antioxidant defenses) and damage (injury biomarkers) were studied in both groups of patients (Figure 5). MTX + ozone increased the capacity of the endogenous antioxidant system to counteract oxidative damage, resulting in a significant decrease (*p* < 0.05) in damage to biomolecules (lipids, MDA and proteins, and AOPP), as well as in TH levels and nitric oxide concentrations. By contrast, patients who did not receive ozone showed a reduction in their antioxidant defenses and a higher level of damage (Figure 5A,B). To find out whether there was any relationship between redox markers and clinical variables, the correlations between these variables were evaluated, only where correlations could be found after treatment with MTX + medical ozone; this suggests a stabilization of the antioxidant/prooxidant balance in the patients. GSH was the only protective redox marker that correlated (*p* < 0.05) with all clinical variables (GSH vs. CRP = 0.68, VCG = 0.63, DAS28 = 0.57, and HAQ-DI = 0.72), whereas SOD correlated with acute-phase reactants, and the Disability Index suggested its participation in the inflammatory process (SOD vs. CRP = 0.5, VCG = 0.51, and HAQ-DI = 0.61).

MTX has been associated with hepatotoxicity in some patients. Elevated transaminase levels and increased liver stiffness were observed in 20 patients out of a sample of 108. Transaminase levels and liver stiffness are early markers of liver fibrosis [45].

In addition to the clinical efficacy observed for the combined therapy of MTX + medical ozone, the hepatoprotective effects of ozone were added in a model for hepatotoxicity induced by carbon tetrachloride, increasing antioxidant defenses (SOD, CAT, and GSH) and reducing injury markers (lipid peroxidation), thus preserving the concentrations of calcium-dependent ATPase, glucose-6-phosphate dehydrogenase, hepatic glycogen, and phospholipase A.

Likewise, the levels of transaminases and cholinesterase were reestablished with less general liver damage and a decrease in the area damaged by lipidosis [14].

The clinical study in patients with RA showed that treatment with the combined therapy MTX + ozone reduced the risk of hepatotoxicity compared to monotherapy [45]. Similar results were found by other authors, where the protective effects of ozone against MTX-induced hepatotoxicity in rats [46,47] were studied.

In order to clarify whether there was any relationship between reduced glutathione and GGT activity in the patients, correlations between both variables were evaluated. In total, 70% of RA patients displayed an inverse lineal correlation (*r* = −0.61, *p* = 0.013). No side effects were observed, nor was MTX withdrawal necessary during the prospective study [44,46].

## 4. Discussion

The results of the experimental models developed and applied have shown that medical ozone “per se” exhibited beneficial effects in diseases involving cartilage damage and bone remodeling. The clinical studies corresponded to the experimental findings in animals.

### 4.1. MTX and Adenosine Mechanisms in Rheumatoid Arthritis

Methotrexate is a structural analogue of folic acid. It was initially introduced to the market as an anticancer agent. In the treatment of RA, it was used at low doses (15–25 mg/week) in the mid-1980s. Although its mechanism of action is not fully understood, it is still considered a first-line drug for RA. Various hypotheses have been considered to explain the beneficial effects of MTX in RA, including folate antagonism, decreased adhesion molecules, polyamine inhibition, and others. Currently, adenosine signaling is the most widely accepted rationale for explaining the mechanism of MTX in RA. This is because MTX increases adenosine levels, activates extracellular receptors, and promotes a complete anti-inflammatory state [48]. Thus, adenosine is an essential mechanism in the anti-inflammatory actions of MTX [49].

Under physiological conditions, adenosine is found at very low levels due to its metabolism, but in situations of stress such as hypoxia, inflammation, tissue damage, and pain, its concentrations rise appreciably. Along with the anti-inflammatory effects of adenosine-mediated signaling, its immunosuppressive actions have been referred to [25].

Adenosine is a low-molecular-weight mediator that regulates multiple physiological and pathological cellular functions. Its actions result from the activation of its receptors (A1, A2A, A2B, and A3) mediated by G proteins. It is generated by the dephosphorylation of ATP, ADP, and AMP to adenosine. Once formed, it is metabolized by the enzyme adenosine deaminase (ADA), which plays a pathological role in RA by inducing chondrocyte death and synoviocyte proliferation, with impairs the differentiation of mesenchymal stem cells into osteoclasts and mineralization [50,51].

The receptor subtype involved in adenosine signaling in RA is still a matter of controversy. Reference is made to the A2A and A3 receptors. Other authors refute the involvement of A3 receptors [50,52], while the activation of A1, in conjunction with A2A, is left open.

It is now accepted that adenosine acts as an immunosuppressant and contributes to immune tolerance. Alterations in its signaling mechanisms, as well as modifications in the expression of its receptors and enzymes, can lead to the development and maintenance of autoimmune diseases such as RA [25].

### 4.2. Medical Ozone and Adenosine Mechanisms in Rheumatoid Arthritis

Synovitis is a critical pathological event preceding the clinical onset of RA [21]. The anti-inflammatory effects of ozone were mediated by the activity of adenosine A1 receptors, a reduction in oxidative damage to proteins (AOPPs and Fructosylamine), and lipid peroxidation (MDA). A decrease in the inflammatory process is probably the most important goal in the therapy of synovitis. mRNA and proteins for A_1_, A_2a_, A_2b_, and A_3_ adenosine receptors are expressed in human synoviocytes. The results show that the adenosine A1 receptor is involved in the anti-inflammatory effects of medical ozone in carrageenan-induced synovitis in Wistar rats. These results agree with the disappearance of anti-inflammatory effects and the protection provided by ozone against oxidative damage when the specific antagonist of adenosine A1 receptors (DPCPX) was tested (Figure 2). These results corresponded with those found in a carrageenan-induced inflammation model, where reductions in thermal and mechanical hypersensitivity dependent on adenosine A1 receptors were reported [53]

Previous results have shown that adenosine receptor A1 is associated with the protective effects of ozone. In damage by ischemia/reperfusion of the liver, the activation of A1 receptors with CCPA (2-chloro-N6-cyclopentyladenosin)—a specific agonist of the A1 receptors—corresponded to a reduction in transaminases, whereas blocking these receptors by DPCPX increased liver damage. In addition, transaminase and adenosine deaminase levels were reduced in the group treated with ozone, similarly to the group that received the A1 receptor activator (CCPA), confirming the functional relationship between ozone and adenosine A1 receptors [31].

In another study, ozone increased the latency of the first convulsive crisis and restored cell redox balance in pentylenetetrazol-induced seizures (PTZ) in mice. DPCPX completely abolished the protection provided by ozone, which evidenced the role of adenosine receptors A_1_ against brain damage [34]. The anti-inflammatory effects of ozone, mediated by adenosine A_1_ receptors, can be the consequence of a transferable oxidative stress induced by ozone. By promoting a generation of ROS, light and transient, ozone regulates cell redox balance and can represent a stimulus for the expression of A_1_ adenosine receptors. Advance Oxidation Protein Products are markers of oxidative damage to proteins, and an increase in their levels in patients with AR has been observed. In addition, synoviocytes such as fibroblasts (FLSSs) are related to oxidative stress. An exposure of FLSSs to AOPPs regulated the mRNAs and the expression of proinflammatory cytokines, metalloproteinases of the matrix, and the growth factor of the vascular endothelium in a concentration-dependent manner. The degradation of IκB and the nuclear translocation of NF-κB-P65, induced by AOPPs, were blocked significantly by antioxidant activity, such as superoxide dismutase, N-acetyl-L-cysteine, and NADPH oxidase inhibitors. Medical ozone was able to maintain AOPP concentrations at the level of the control group (saline).

Lipid peroxidation leading to the formation of protein adducts promotes proinflammatory responses characterizing a variety of chronic conditions [54]. Malondialdehyde (MDA) is one of the ubiquitous products involved in disease pathogenesis, and its levels are increased in synovial fluid. MDA was significantly reduced by ozone treatment with respect to all experimental groups studied, suggesting that the decrease in hydroxyl radicals available initiated and triggered the peroxidative damage of cell membranes, also suggesting a decrease in RA-associated events mediated by this aldehyde (Figure 1).

Medical ozone exerted beneficial effects on local inflammation induced by carrageenan, and these results were corroborated in the chronic models of RA. Simultaneously, other redox markers were also studied (Table 1). PG/PS and PG/PS + oxygen exhibited low SOD activity, whereas PG/PS + Ozone and control (−) showed no significant differences. The accumulation of superoxide radicals in the presence of overproduced NO leads to the formation of peroxynitrite, a known cytotoxin. Modest increases in superoxide and NO (10-fold in each case) will increase peroxynitrite formation 100-fold. Under proinflammatory conditions, such as in RA, the simultaneous production of superoxide and NO (1000-fold) will increase peroxynitrite formation 1,000,000-fold [55].

It should be noted that MTX halted the progression of the disease—this represents an important therapeutic response. However, there were no significant differences in the group of patients receiving MTX only, when comparing the beginning and the end of the study. By contrast, patients treated with MTX + ozone combined therapy significantly improved (*p* < 0.05) in the context of both clinical variables and cellular redox status. This suggests that—in the context of monotherapy (MTX)—the combination of MTX + ozone enhances clinical response and exerts an additive or synergistic effect on the response of patients with RA, as included in this study. These effects also suggest that they are the result of the contribution made by each of the components of the combined therapy (MTX and ozone) in the control of the disease, given that both agents share common therapeutic targets, such as redox balance and actions associated with adenosine.

**Figure 6:**
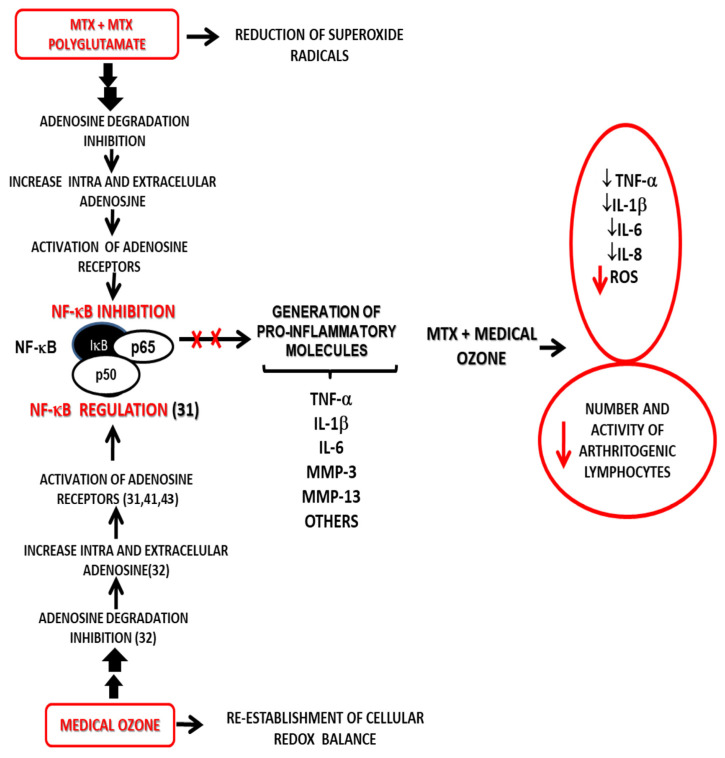
Probable mechanism of action for the combined MTX + ozone therapy integrating both the experimental results and the clinical response of patients suffering from RA—this explains the beneficial effects from the introduction of medical ozone in pharmacotherapeutic treatment [31,32,41,43].

Taken together, MTX, medical ozone, and adenosine results allow us to propose a new mechanism of action explaining both the results obtained in disease models and the clinical response of patients in RA (Figure 6).

The proposed mechanism is based on adenosinergic participation, common to MTX and medical ozone, as essential mechanisms in the treatment of RA—these are capable of regulating the redox state, the production of proinflammatory cytokines, and arthritogenic lymphocytes.

## 5. Conclusions

In an integrative analysis of the results of the present study, the mechanism of action proposed by the authors is found to be the principal aspect in the administration of the combined therapy, as described by the authors. Both (MTX and ozone) share common molecular events, which explains the significant improvement in the clinical response where patients were treated with MTX + ozone. These results are expressed by the different studies carried out at a preclinical level, such as (a) the participation of A_1_ adenosine receptors in the anti-inflammatory and analgesic effects of medical ozone, (b) the reduction in the expression of proinflammatory cytokines, and (c) the regulation of the cellular redox state. These benefits of combined therapy are expanded when a reduction in the risk of hepatotoxicity of MTX + ozone is additionally demonstrated in patients with RA in comparison with monotherapy.

## 6. Future Directions

Medical ozone is capable not only of stopping the progression of RA, but also improving clinical and redox indicators in these patients. The pleiotropy of medical ozone increases its therapeutic efficacy in diseases of oxidative etiology, which means that its efficacy/effectiveness can be hypothesized in patients with RA detected at an early stage (<1 year). Hence, even if they are carriers of this autoimmune disease, they remain asymptomatic, with a good quality of life and without the adverse reactions of the drugs otherwise used to control this immune dysfunction. Further research in this field is currently being developed by our own research group.

## Figures and Tables

**Table 1:** Essential articles by the authors of this manuscript; analyzed and used in the presented results.

Medical Ozone Effects	Brief Study Description	Reference
**Model 1 of Synovitis (acute)**	BALB/c mice. Synovitis inducer: Carrageenan. Specific A1 receptor antagonist (DPCPX), redox status. Optical microscopy. Ozone 15 intra-articular treatments (50 µg/mL).	Oru, GT et al. (2018) [29]
**Model 2 of Arthritis(chronic)**	Lewis rats. Inducer PG/PS. TNF-α, IL-1β mRNA, redox status. Optical microscopy. Ozone for 3.5 weeks (3 times/week), intra-articular ozone (20 µg/mL).	J Dranguet et al. (2013) [30]
**Model 3 of Liver ischemia/Reperfusion**	Wistar rats. 90 min ischemia/90 min reperfusion. Transaminases, specific A1 adenosine receptor agonists (CCPX) and antagonists (DPCPX). Redox status. Optical microscopy. Ozone 15 treatments (50 µg/mL).	León et al. (2008) [31]
**Model 4 of Liver Ischemia/Reperfusion**	Wistar rats. 90 min ischemia/90 min reperfusion. Transaminases, ATP degradation, adenosine production. Redox status. Ozone 10 treatments (50 µg/mL).	C Peralta et al. (2000) [32]
**Model 5 of Hepatotoxicity by CCl_4_**	Wistar rats. Hepatotoxicity (hepatic damage markers). Optical microscopy. Redox status. Ozone 15 treatments (50 µg/ml).	OS León et al. (1998) [14]
**Model 6 of Alcohol Withdrawal**	Wistar rats. Ethanol withdrawal. Redox status. Behavioral tests. Ozone 10 treatments (20 µg/mL).	MT Díaz Soto et al. (2012) [11]
**Clinical Study: Diabetes and Diabetic Foot**	100 patients (52 received ozone). Healing, diabetic control, redox status. Ozone 20 treatments (50 µg/mL).	G Martínez-Sánchez et al. (2005) [12]
**Clinical Study: Elderly Patients**	60 patients (45 received ozone) with rheumatoid arthritis and bronchial dysfunctions. Disease activity, prostaglandins (TBX A2, PGI2), redox status. Ozone 20 treatments (20-30 µg/mL).	OS, León et al. (2022) [13]
**Clinical Study: Meta-analysis on Ozone Safety via Systemic Routes**	657 patients. Rectal insufflation and major autohemotherapy. According to Evidence-Based Medicine standards.	R Viebahnn, et al. (2016) [27]
**Clinical Study: MTX + Ozone in Rheumatoid Arthritis**	60 patients (30 received ozone). Disease activity, redox status, antibodies vs. cyclic citrullinated peptides.	OS, León et al. (2016) [33]
**Clinical Study: Hepatotoxicity Risk in MTX + Medical Ozone**	100 patients (50 received ozone). Disease activity, redox status, antibodies vs. cyclic citrullinated peptides. Hepatic damage markers. Hepatotoxicity risk evaluation. Ozone 20 treatments (20–40 µg/mL).	Oru, GT et al. (2017) [34]

**Table 2:** Mean and 95% Confidence Intervals of mRNA of IL-1β and TNF-α in PG/PS-induced arthritis.

		95% Confidence Interval	
MARKERS	MEAN ± SD	LOWER LIMIT	UPPER LIMIT
**IL-1β**			
PG/PS (inductor del daño)	2.81 ± 0.04	2.76	2.86
PG/PS + Ozone	1.96 ± 0.08	1.85	2.07
PG/PS + Oxygen	2.87 ± 0.04	2.82	2.92
**TNF-** **α**			
PG/PS (inductor del daño)	3.9 ± 0.22	3.6	3.9
PG/PS ± Ozone	2.75 ± 0.22	2.45	3.05
PG/PS + Oxygen	4.22 ± 0.43	3.63	4.81

**Table 3:** Redox marker levels and confidence interval in spleen homogenates: (**A**) carrageenan-induced synovitis and (**B**) PG/PS-induced arthritis.

		95% Confidence Interval	
MARKERS	MEAN ± SD	LOWER LIMIT	UPPER LIMIT
**(A)** **. Acute Model**			
**MDA** **(μM)**			
Control (saline)	1.6 ± 0.001(a)	1.6	1.61
Carrageenan	1.99 ± 0.01(b)	2.1	2.2
Carrageenan + Ozone	1.4 ± 0 (c)	1.4	1.4
Carrageenan + Oxygen	1.8 ± 0.06 (d)	1.73	1.89
**TH (μM)**			
Control (saline)	49.3 ± 4.4 (a)	37	49.4
Carrageeenan	57 ± 2.8 (b)	48	56
Carregenaan + Ozone	39.6 ± 0.07 (c)	39	39.5
Carrageenan + Oxygen	45.4 ± 0.3 (a)	45	46
**(B)** **. Chronic Model**			
**SOD** ** (U/mg protein)**			
Control (saline)	25.8 ± 1.32 (a)	24.5	27
PG/PS	3.7 ± 1.45 (b)	2.7	5.2
PG/PS + Ozone	33.6 ± 1.39 (a)	33.6	36.4
PG/PS + Oxygen	11.3 ± 1.75 (c)	9.5	13
**CAT (U/mg protein)**			
Control (saline)	212.2 ± 13.8 (a)	193	231
PG/PS	182 ± 0.001 (a)	181	182
PG/PS + Ozone	391 ±2.6 (b)	387	394
PG/PS + Oxygen	208 ± 19.6 (a)	178	236

Legend: TH, total hydroperoxides; MDA, malondialdehyde; SOD, suproxide dismutase activity; CAT, catalase activity; PG/PS, glycan/polysaccaride peptide. Data represent the mean ± SD of each group (*n* = 5). The different letters indicate significant differences (*p* < 0.05) between groups.

**Table 4:** Clinical variables of patients with RA at the beginning and at the end of the study.

	MTX (*n* = 30)		MTX + Ozone (*n* = 30)	
Clinical Variables	Start	End	Start	End
Pain	8.2 ± 0.47 ^(a)^	7 ± 0.65 ^(a)^	9.2 ± 0.37 ^(a)^	4.7 ± 0.33 ^(b,c)^
DAS28	5.64 ± 0.36 ^(a)^	5.21 ± 0.37 ^(a)^	6.4 ± 0.22 ^(a)^	3.2 ± 0.37 ^(b,c)^
HAQ-DI	1.53 ± 0.16 ^(a)^	1.14 ± 0.17 ^(a)^	1.80 ± 0.10 ^(a)^	0.75 ± 0.07 ^(b,c)^
CRP (mg/L)	21.08 ± 7.12 ^(a)^	13.14 ± 4.26 ^(a)^	16.2 ± 4.75 ^(a)^	5.53 ± 1.48 ^(b,c)^
ESR	40.9 ± 6.62 ^(a)^	40.3 ± 6.08 ^(a)^	36.7 ± 6.38 ^(a)^	20 ± 4.64 ^(b,c)^
Anti-CCP (U/mL)	102.8 ± 34 ^(a)^	119.2 ± 39 ^(a)^	107 ± 62 ^(a)^	89.7 ± 33 ^(b,c)^

Legend: MTX group: Methotrexate + Ibuprofen + Folic acid. MTX + ozone group; same as MTX group + medical ozone: DAS_28_. Disease activity (low 3.2; moderate 3.2 and 5.1; high 5.1. HAQ-DI, Disability Index Questionnaire (+1.25); CRP, “C” Reactive Protein (+6 mg/L in serum); ESR, Erythrocyte Sedimentation Rate (male 7–8 mm, female 11–16 mm); Anti-CCP, antibodies against Cyclic Citrullinated Peptides (10 U/mL in serum). “Start” stands for the begin of the study and “End” for termination of the final ozone treatment 21 days after “Start”. All data shown represent mean ± SD. Mean values with different letters indicate significant differences (*p* < 0.05) (c) (*p* < 0.05) on day 21 (at the end of the study) “MTX + ozone group” vs. “MTX group” [44].

## Data Availability

All data used are cited in this article.

## Abbreviations

A1AR	A1 adenosine receptors
ADA	Adenosine Deaminase
Anti-CCP	Antibodies against Cycllic Citrullinate Peptide
AOPP	Advanced Oxidation Protein Products
bDMARDs	Biological DMARDs
CAT	Catalase
CCPA	2-chloro-N6-cyclopentyladenosine
CENIC	National Center for Scientific Research
CRP	C Reactive Protein
csDMARDs	Conventional synthetic DMARDs
DAS28	Disease Activity Score 28
DMARDs	Disease Modifying Antirheumatic Drugs
DPCPX	8 cyclopentyl-1,3 dipeptilxanthine
ESR	Erythrocyte Sedimentation Rate
FLSS	Fibroblasts like synoviocytes
GSH	Reduced Glutathione
GSH-Px	Glutathione Peroxidase
H2O2	Hydrogen Peroxide
HAQ-DI	Assessment Questionnarie-Disabiity
HCLO	Hypochlorous Acid
I/R	Ischemia/Reperfusion
IL-1β	Interleukin 1 Beta
IL-6	Interleukin 6
IL-8	Interleukin 8
LPO	Lipid Peroxidation
MDA	Malondialdehyde
MMP	Metalloproteinases
MPO	Mieloperoxidase
MTX	Methotrexate
NF-κB	Nuclear Factor kappa B
NO	Nitrric Oxide
Nrf2	(Erythroid-derived 2)-like 2
O_2_	Oxygen
O_2_^−^	Superoxide Radical
O_3_	Ozone
O_3_/O_2_	Ozone/Oxygen mixture
OS	Oxidative Stress
p50	P50 subunit of NF kappa B
P65	P65 subunit of NF kappa B
PG/PS	Glycan/Polysaccaride Peptide
PGI2	Prostacyclin 2
RA	Rheumatoid Arthritis
ROS	Reactive Oxygen Species
SOD	Superoxide Dismutase
TB	Tuberculosis
TBX A2	Thromboxane A2
TH	Total Hydropeoxides
TNF-α	Tumor Necrosis Factor alpha
tsDMARDs	Targeted synthetic DMARDs
VEGF	Vascular Endothelial Growth Factor
